# Radioluminescence microscopy of tumor organoids enabled by a reconfigurable high-resolution imaging setup

**DOI:** 10.1186/s13550-026-01484-y

**Published:** 2026-07-07

**Authors:** Sofia Demianova, Martin Dierolf, Maximilian Reichert, Laura Schmidleitner, Franz Pfeiffer, Wolfgang A. Weber

**Affiliations:** 1 Chair of Biomedical Physics, Department of Physics, TUM School of Natural Sciences, Technical University of Munich, Garching, Germany; 2 Munich Institute of Biomedical Engineering (MIBE), Technical University of Munich, Garching, Germany; 3 TUM Institute for Advanced Study, Technical University of Munich, Garching, Germany; 4 Department of Nuclear Medicine, TUM University Hospital, Klinikum rechts der Isar, Munich, Germany; 5 Center for Translational Pancreatic Cancer Research, TUM School of Medicine and Health, Clinical Department for Internal Medicine II, TUM University Hospital, Munich, Germany; 6 Center for Organoid Systems (COS), Technical University of Munich, Garching, Germany; 7 Bavarian Cancer Research Center (BZKF), Munich, Germany

**Keywords:** High-resolution imaging, Instrumentation, Radioluminescence microscopy, Radiotracer imaging, Tumor organoids

## Abstract

**Background:**

This study aims to construct and implement an experimental radioluminescence microscopy (RLM) setup using a standard light microscope to visualize tumor organoids with high resolution. This approach seeks to address the challenges of imaging organoids, facilitating advancements in precision medicine for cancer research. Tumor organoids grown in collagen were incubated with 100 MBq to 120 MBq of 18F-fluorodeoxyglucose to visualize metabolic activity within their structures. Imaging was performed using a sensitive electron-multiplying charge-coupled device in conjunction with a scintillator crystal and an optical system with a high light collection efficiency and adapted magnification.

**Results:**

The constructed RLM setup demonstrated high-resolution imaging of organoid samples, significantly exceeding the resolution achievable with positron-emission tomography. The combination of RLM with brightfield and fluorescence microscopy provided comprehensive morphologic and metabolic characterization of the organoids at sub-organoid spatial resolution.

**Conclusion:**

The study successfully illustrates the feasibility of using a modified light microscope for RLM to achieve imaging of tumor cell metabolism in organoids with a spatial resolution on the order of tens of micrometers. This approach opens new avenues for studies of tumor cell metabolism during tumor progression and during therapeutic interventions.

## Introduction

Tumor heterogeneity, marked by variations within tumors, is linked to metastasis, relapse, and resistance to therapy, which are significant contributors to treatment failures and poor prognosis. This heterogeneity spans multiple levels, including genetic, epigenetic, metabolic, and microenvironmental variability, all of which can influence treatment response. Among these, spatial heterogeneity of tumor metabolism is of particular relevance for nuclear medicine imaging, as it directly affects the interpretation of radiotracer uptake patterns. This highlights the urgent need for personalized tumor models that accurately reflect the individual heterogeneity of patients’ tumors, crucial for advancing precision medicine [1].

Tumor organoids have emerged as a promising class of cancer models that, unlike standard cell cultures, replicate the complex three-dimensional structure of human cancer. This makes them valuable tools for investigating tumorigenesis and cancer progression in vitro, holding significant promise for translational studies [2–5].

Accelerated glucose utilization is a hallmark of many malignancies and forms the basis for the widespread clinical use of positron-emission tomography (PET) with the glucose analog 18F-fluorodeoxyglucose (FDG) for cancer detection and staging [6]. In addition, inhibition of glucose metabolism is being explored as an anticancer therapeutic strategy. A detailed understanding of the FDG-PET signal and the effects of metabolic therapies would greatly benefit from data on the microscopic heterogeneity of metabolic processes. The inherent limitation of PET to a low resolution in the millimeter range presents a challenge for imaging organoid samples [7–9]. Alternative techniques, such as autoradiography, can achieve high spatial resolution, including cellular and subcellular localization, but typically require ex vivo sample processing and are not compatible with preserving live tissue architecture. In contrast, flow cytometry-based approaches such as radioFACS enable quantitative single-cell analysis but require tissue dissociation, thereby losing spatial context [9, 10].

Recent advancements have enabled the imaging of PET tracers in 2D cell cultures with single-cell resolution using radioluminescence microscopy (RLM) [11]. RLM employs a scintillator material (normally an inorganic crystal) placed in contact with the radiolabeled sample to convert ionizing radiation directly into detectable visible light, facilitating precise 2D imaging of the radionuclide distribution in living cells. RLM can be used for cell metabolism characterization, e.g., using FDG-labeled samples, cell proliferation estimation, and drug binding studies using single frames or the kinetic mode, with each acquisition happening at regular intervals [12].

Different implementations of RLM have been proposed in the literature. For instance, Sung et al. demonstrated radioluminescence imaging using an upright microscope configuration [13]. These systems typically employ optical demagnification to increase the amount of scintillation light collected per detector pixel, thereby improving signal-to-noise characteristics. In that work, as well as in related systems [11, 12, 14], a short focal-length tube lens was employed to achieve optical demagnification, thereby increasing the amount of emitted scintillation light collected per detector pixel and improving sensitivity. In contrast, other approaches rely on custom-built optical systems optimized for low-light imaging, which may require specialized alignment and instrumentation expertise.

This study presents a proof of concept for applying RLM to organoids using a conventional microscope. In contrast to previous approaches that relied on custom-built equipment [14], this makes the imaging setup easily constructable and accessible, particularly for clinical facilities in nuclear medicine. Furthermore, this work introduces a newly developed protocol for the sample preparation, specifically optimized for compatibility with RLM. When integrated with bright-field (BF) and fluorescence microscopy, RLM enables multimodal imaging that combines structural and metabolic information. While the detected FDG signal primarily reflects cellular metabolic activity and local microenvironmental conditions, it is not inherently cell-type specific. However, correlation with fluorescence markers provides a pathway for linking radiotracer uptake to biological features within the organoid.

Overall, the presented approach addresses key limitations of existing imaging techniques by enabling high-resolution radiotracer imaging of three-dimensional tumor models in an experimentally accessible setup, thereby supporting future studies of metabolic heterogeneity and treatment response in organoid systems.

## Materials and methods

### Radioluminescence microscope design

The development of the RLM setup was guided by the following key requirements:

- Scintillator material with emission wavelength matching the detector sensitivity
- Optical system with high light collection efficiency and suitable magnification
- Sensitive detector for low-light imaging
- Availability of multimodal imaging (bright-field, phase-contrast, fluorescence)
- Appropriate sample handling and positioning strategy

A Zeiss Axiovert 135 inverted microscope (Zeiss, Oberkochen, Germany) was selected as the base platform due to its modular design and support of bright-field, phase-contrast, Differential Interference Contrast (DIC), and fluorescence microscopy. The microscope was additionally equipped with a FITC filter set (excitation: 475 nm, emission: 530 nm, Zeiss), matching the mNeonGreen fluorescent label used for organoid samples. A C-mount camera adapter (60-C 1" 1,0$\times$ 456105, Zeiss) was used to connect the detector.

To maximize light collection efficiency, a Zeiss Plan-Apochromat 10$\times$/0.45 objective was used. The achievable imaging depth is limited by both the working distance of the objective and the requirement for close proximity between the radioactive sample and the scintillator surface. As a result, optimal imaging conditions are achieved for organoids with typical dimensions in the sub-millimeter range, or in configurations where the distance between the radiotracer distribution and the scintillator is minimized.

**Fig. 1:**
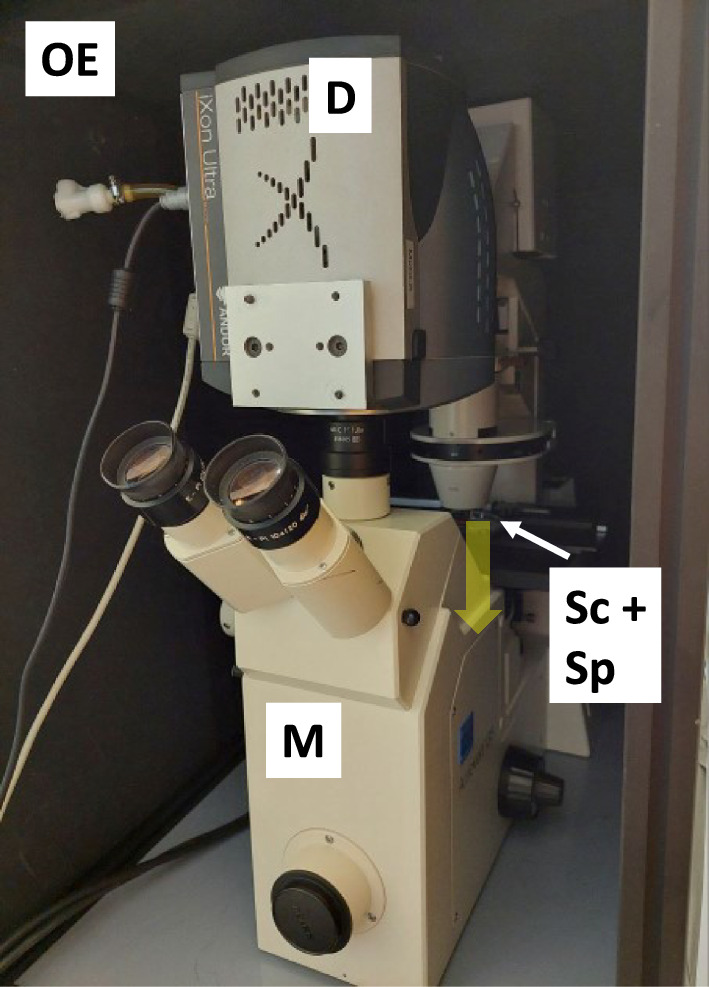
Photo of the implemented radioluminescence microscope setup. D: Detector; OE: Optical Enclosure; M: Microscope; Sc: Scintillator; Sp: Specimen. The yellow arrow indicates the light emission direction

The entire microscope was placed inside a custom-built, light-tight enclosure constructed from Thorlabs components (Thorlabs, Bergkirchen, Germany) to minimize background signal from stray light. The final RLM setup is presented in Figure 1.

Image acquisition was performed using an Andor iXon Ultra 897 electron-multiplying charge-coupled device (EMCCD) camera (Oxford Instruments, Abingdon, UK), connected to a control computer via USB 2.0 and operated using the Andor Solis software. The detector was operated with deep cooling down to −65 $^{\circ }\text {C}$ using the built-in air cooling system. The main technical specifications of the camera are summarized in Table 1.

**Table 1:** Andor iXon Ultra 897 key specifications

Active pixels	512$\times$512
Pixel size (µm²)	16$\times$16
Image area (mm²)	8.2$\times$8.2
Read-out noise	$\le 1\,e^{-}$RMS with EM gain
QE max.	$\ge 95$% @ 480–690 nm.
Chip temperature (with air cooling)	−65 $^{\circ }\text {C}$

During radioluminescence imaging, an EM gain of up to 300 was used. Pixel binning with a factor of 4 was applied to increase signal sensitivity. For phase-contrast and fluorescence imaging, the EM gain was disabled.

Phase-contrast images were acquired with the filter block removed using the standard microscope illumination. For fluorescence imaging, the main illumination was turned off and a Zeiss 12 V/100 W halogen lamp was used together with the FITC filter set. Both phase-contrast and fluorescence images were acquired with an exposure time of 0.01 seconds. Radioluminescence images were acquired with an exposure time of 300 seconds.

A CdWO_4_ scintillator crystal (10 mm diameter, 500 $\upmu$m thickness) was used due to its high light yield (12000 photons/MeV to 15000 photons/MeV), high effective atomic number (Z=64), high density (7.9 g/cm$^{3}$), and emission spectrum centered around 475 nm, matching the detector sensitivity.

The scintillator crystal was placed at the bottom of a Petri dish. For in situ imaging, collagen gel containing organoids was carefully transferred from the culture wells to the Petri dish using a spatula, minimizing disruption of the matrix structure. During imaging, samples were maintained in a thin layer of Phosphate-Buffered Saline (PBS, Thermo Fisher Scientific) sufficient to prevent drying while ensuring close contact with the scintillator.

For ex situ imaging after collagen digestion, the organoids were directly pipetted onto the scintillator surface.

Focusing was performed by identifying the top and bottom surfaces of the scintillator crystal in bright-field mode. The imaging plane was set to the top surface of the crystal, corresponding to the plane closest to the sample.

### Organoid preparation and radiolabeling

Murine pancreatic ductal adenocarcinoma (PDAC) organoids expressing mNeonGreen (#9591-mNG) were used in this study. The organoids were derived from tumor cells (line 9591) originally isolated from a genetically engineered mouse model of pancreatic cancer (Ptf1aCre/+; KrasG12D/+; KC mouse) as previously described [15].

Organoids were generated and cultured in floating collagen I gels following established protocols [5, 16]. Briefly, cells were embedded in collagen I matrices and maintained under standard organoid culture conditions. Detailed characterization of this organoid model, including genetic background, cellular composition, and phenotypic heterogeneity, has been reported previously [5, 16].

Initial experiments were performed on fixed organoids to optimize imaging parameters. Subsequently, all FDG uptake experiments were conducted on viable (non-fixed) organoids to assess biologically relevant radiotracer accumulation.

For FDG incubation, organoids were starved in 500 ${\upmu }$L glucose-free Dulbecco’s Modified Eagle Medium (DMEM, Thermo Fisher Scientific) for 45 minutes, followed by incubation with FDG (total activity 100 MBq to 120 MBq) for 1.5 hours. The applied activity concentration was approximately 200 MBq/mL to 240 MBq/mL (calculated based on a total activity of 100 MBq to120 MBq in 500 ${\upmu }$L incubation volume).

**Fig. 2:**
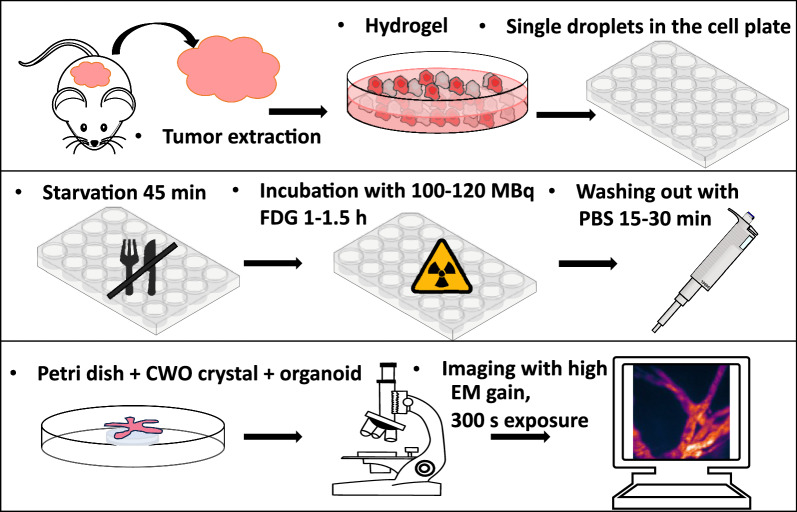
Schematic overview of the complete organoid preparation, radiolabeling, and imaging workflow

After incubation, the medium was removed and the samples were washed in PBS for 15–30 minutes. The process is schematically presented in Figure 2.

**Fig. 3:**
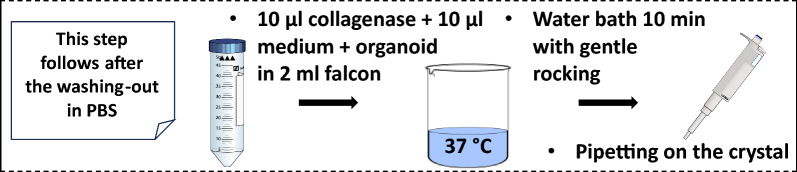
Additional collagen digestion step introduced in this work to obtain organoids imaged ex situ. After standard radiolabeling, the collagen matrix is enzymatically dissolved using collagenase, allowing the organoid to be placed directly onto the scintillator prior to imaging

To reduce the distance between the sample and the scintillator, two alternative preparation strategies were employed. In the first approach, organoids were cultured in a reduced collagen volume of 100 $\upmu$L instead of 200 $\upmu$L. In the second approach, the collagen gel was digested using collagenase. For this purpose, collagenase was mixed 1:1 with the sample medium, and the radiolabeled organoid was incubated in this solution at 37 $^{\circ }\text {C}$ for 10 minutes. The digested sample was then directly pipetted onto the scintillator crystal and imaged without further washing. This additional step is illustrated in Figure 3.

All images were processed using Python-based analysis tools. For resolution assessment, intensity profiles were extracted along fine structural features visible in both radioluminescence and fluorescence images. The Full Width at Half Maximum (FWHM) of these profiles was calculated and used as a metric for spatial resolution. Relative resolution was estimated by comparing the FWHM values obtained from radioluminescence and fluorescence images.

Background contributions, including detector dark signal, were subtracted during image processing. All images were acquired under the same imaging conditions.

## Results

### Setup design assessment

Cooling the EMCCD detector to −65 $^{\circ }\text {C}$ resulted in a stable low-noise imaging environment. The use of a custom-built light-tight enclosure and operation in a dark room minimized the contribution of stray light. This was verified by comparing images acquired with the detector shutter open and closed, demonstrating negligible background signal contribution.

The influence of the optical configuration on signal intensity was evaluated. The 10$\times$/0.45 NA objective provided higher signal brightness compared to lower-NA alternatives.

Focusing was found to have a strong impact on both signal brightness and spatial localization. Images acquired while focusing on the top surface of the scintillator crystal showed the highest contrast and signal intensity. The application of 4$\times$ binning further increased the detected signal intensity.

### Influence of sample preparation and image resolution

**Fig. 4:**
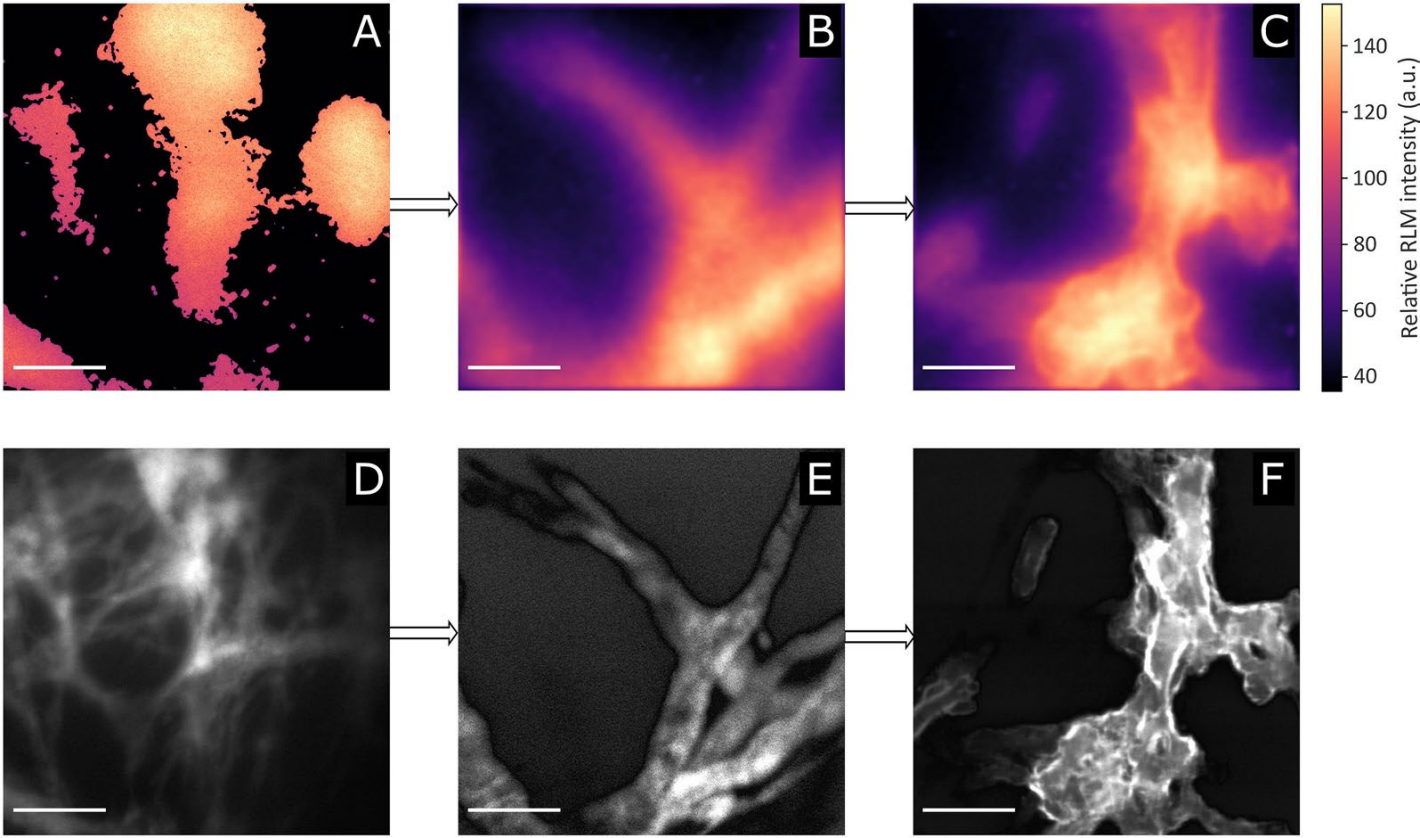
Comparison of different organoid preparation approaches for radioluminescence imaging. Radioluminescence (RLM) images are shown in panels (**A**–**C**), and the corresponding fluorescence (FL) images of the same organoids are shown in panels (**D**–**F**). (A,D) Organoid imaged *in situ* in 200 $\upmu$L collagen gel. (**B,E**) Organoid imaged *in situ* in reduced collagen volume of 100 $\upmu$L. (**C,F**) Organoid imaged *ex situ* after complete collagen digestion using collagenase. A clear improvement in spatial resolution is observed when reducing the collagen thickness and after complete gel removal. The fluorescence channel provides structural contrast and delineates the boundaries of the organoids, enabling interpretation of the spatial distribution of FDG uptake observed in the RLM images. In panel (A), a more diffuse signal and background subtraction lead to contrast discontinuities. The RLM signal intensity within the field of view (FOV) is shown in relative units (a.u.). Scale bars: 200 $\upmu$m.

Radioluminescence imaging was first performed on organoids grown *in situ* in 200 $\upmu$L collagen gels. As shown in Figure 4A, although the organoids were detectable, the obtained images exhibited substantial blurring and limited structural detail. The increased noise and diffuse appearance are attributed to the larger distance between the organoid and the scintillator, resulting in reduced photon collection efficiency and increased signal blurring.

To reduce the distance between the sample and the scintillator crystal, organoids were subsequently grown in a reduced collagen volume of 100 $\upmu$L. Under these conditions, normal organoid growth was observed. As shown in Fig. 4B, this approach resulted in improved spatial resolution and higher image sharpness. However, due to the reduced mechanical stability of the thinner gel, sample handling became more challenging and some samples were damaged during transfer.

To further improve spatial resolution, the collagen gel was completely removed using enzymatic digestion with collagenase. This approach resulted in a substantial improvement in image sharpness and allowed fine structural features of the organoids to be resolved, as shown in Figure 4C.

In Figure 4, radioluminescence and fluorescence images are presented side by side to enable visual comparison of tracer uptake and organoid morphology. The color scale applies only to the RLM channel, whereas the fluorescence image serves solely as a structural reference, highlighting the regions of the activity accumulation.

### Spatial resolution analysis

**Fig. 5:**
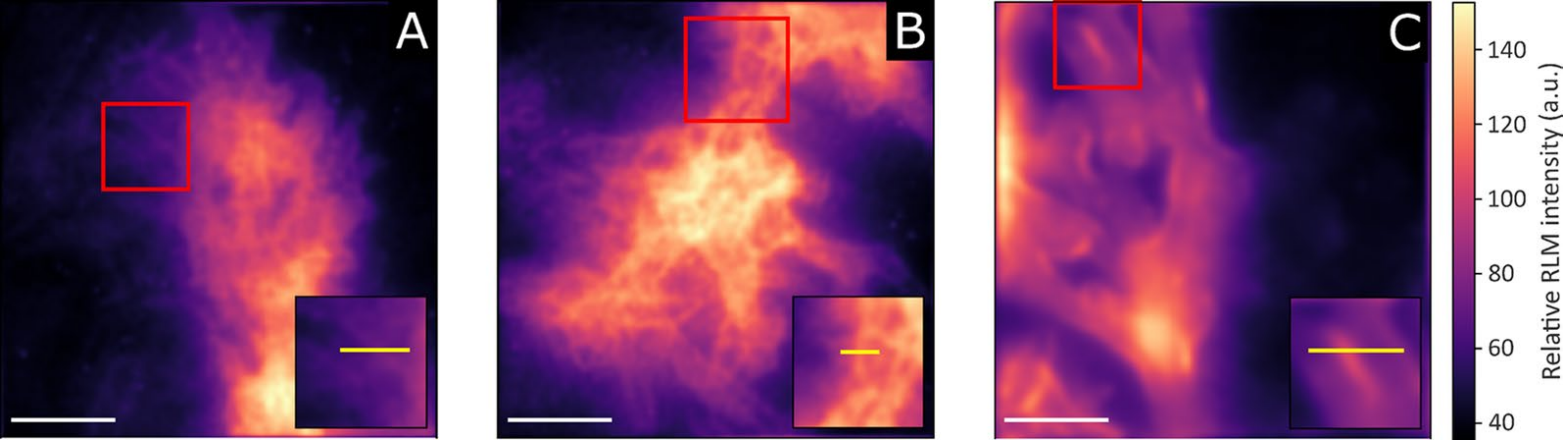
Radioluminescence images of three different *ex situ* organoids imaged after collagen digestion (**A–C**). For each sample, a magnified region of interest is shown together with a yellow line indicating the position used for the intensity profile and FWHM calculation. The RLM signal intensity within the field of view (FOV) is shown in relative units (a.u.). Scale bars: 200 $\upmu$m

To quantify the spatial resolution, radioluminescence images of three different organoids imaged ex situ after collagen digestion were analyzed. Figure 5 shows the corresponding radioluminescence images with magnified regions of interest. For each sample, a line profile was extracted across a fine structural feature and used to calculate the FWHM.

The extracted FWHM values obtained from both radioluminescence and corresponding fluorescence images are summarized in Table 2.

**Table 2:** FWHM values measured for features in radioluminescence and fluorescence images of organoids imaged ex situ after collagen digestion

Sample	FWHM RLM [$\upmu$m]	FWHM Fluorescence [$\upmu$m]	Factor
Organoid A	19.2	19.2	1.0
Organoid B	11.2	8.0	1.4
Organoid C	35.2	22.4	1.57

The radioluminescence signal exhibits a spatially heterogeneous distribution within the organoids, which is consistent with variations in FDG uptake. While the signal appears partially diffuse, this is primarily attributed to physical effects inherent to radioluminescence imaging, including positron range and optical spreading within the scintillator.

The measured FWHM values indicate that the spatial resolution of radioluminescence imaging is on the order of tens of micrometers. This is lower than the resolution of fluorescence microscopy, but sufficient to resolve intra-organoid variations in tracer uptake. The smallest resolved structures were observed close to the practical resolution limit of the system, enabling visualization of internal features and heterogeneous activity distribution within the organoids. The spatial correspondence between radioluminescence and fluorescence images indicates that regions of elevated signal coincide with the structural boundaries of the organoids.

Control experiments on gel parts not containing the samples confirmed that the applied washing procedure effectively removes extracellular tracer, with no significant residual signal detected in the surrounding medium.

### Calibration and sensitivity

Calibration of the RLM signal was performed using established approaches based on imaging of liquid droplets with known activity, as described in previous studies [14].

To extend this approach to the biological system under investigation, additional calibration experiments were performed using FDG-labeled adherent PC3 human prostate cancer cells. All calibration images were acquired under the same imaging conditions (total activity, EM gain, exposure time, and optical configuration) as the organoid measurements. In this case, the total activity on the scintillator was measured using a dose calibrator, while the number of cells within the FOV was estimated from microscopy images. This enabled an approximate correlation between the detected signal and activity. A detailed characterization of signal linearity was not performed and remains beyond the scope of this study.

Despite this improvement, the calibration introduces intrinsic uncertainties, primarily due to incomplete cell adhesion, sample geometry, and the spatial distribution of the radiotracer. An approximate activity per cell can be estimated from these measurements and was used to establish the overall sensitivity and signal scaling of the system. In our experiments, this corresponds to an order of magnitude of approximately 2–30 Bq per cell under the applied labeling conditions. However, this estimate is strongly dependent on experimental factors such as cell adhesion efficiency and washing conditions.

Based on these combined approaches, the detectable activity was estimated to be on the order of several hundred Bq within the FOV, consistent with previously reported radioluminescence microscopy systems [14].

For image visualization, RLM data are presented as relative signal intensity (arbitrary units, a.u.). A direct pixel-wise conversion of signal intensity into local radioactivity is not physically meaningful in radioluminescence microscopy due to intrinsic effects such as positron range and optical spreading within the scintillator.

Consequently, the RLM signal should be interpreted as a system-dependent, semi-quantitative measure. Within a consistent experimental configuration, however, these measurements enable reliable relative comparisons of radiotracer uptake within and between samples.

## Discussion

In this work, an RLM setup based on a conventional inverted light microscope was successfully implemented and validated for imaging tumor organoid specimens. The main objective was to develop an experimentally accessible and robust system that can be readily adopted in a nuclear medicine environment while maintaining sufficient sensitivity and spatial resolution for biologically relevant applications.

Compared to the original radioluminescence microscopy concept introduced by Pratx et al. [11], and the modular low-light microscope described by Kim et al. [12], the present setup follows a more conservative and pragmatic design approach. While Kim et al. proposed a highly modular optical architecture optimized for multiple low-light imaging modalities, such systems require careful optical alignment and dedicated optomechanical expertise. In contrast, the present work demonstrates that a commercially available inverted microscope platform can be successfully adapted for RLM by integrating only a limited number of key components, namely a high-sensitivity EMCCD detector, a high-NA objective, an appropriate filter set, and a CdWO_4_ scintillator crystal. This significantly simplifies implementation and reduces the risk of optical misalignment while preserving sufficient performance for organoid imaging.

More recently, Khan et al. [14] demonstrated a highly advanced positron emission microscopy system for imaging patient-derived tumor organoids. In their work, the organoids were mechanically extracted from the surrounding matrix, a procedure that may introduce structural damage and lead to partial disruption of the three-dimensional architecture, potentially increasing unwanted background noise signal. In contrast, in the present work, the surrounding collagen matrix is enzymatically dissolved, allowing the organoid to be released and transferred onto the scintillator surface without mechanical disruption or fragmentation. This approach enables ex situ imaging of intact organoids and provides radioluminescence images with reduced structural distortion and improved structural interpretability of the radioluminescence signal.

A key result of this work is the demonstration that sample preparation plays a decisive role in determining the achievable spatial resolution in RLM of three-dimensional specimens. Initial in situ imaging in standard 200 $\upmu$L collagen gels resulted in strong signal blurring due to the large distance between the radioactive sample and the scintillator. Reducing the gel thickness to 100 $\upmu$L already led to a noticeable improvement, although at the cost of increased mechanical fragility of the samples.

The most significant improvement was achieved by introducing an additional collagen digestion step using collagenase, which allowed the organoids to be imaged ex situ directly on the scintillator surface. The achieved spatial resolution was on the order of tens of micrometers. While this is lower than the resolution of fluorescence microscopy, it is sufficient to resolve the spatial heterogeneity of tracer uptake within organoids. The apparent signal spreading observed in the images is largely governed by intrinsic physical effects, including positron range and scintillation light propagation, which limit precise localization of the radioactive decay events.

This approach is conceptually related to the strategy used by Khan et al. [14], where close contact between the specimen and the detector is also essential for achieving high resolution. However, the present work demonstrates that a similar principle can be realized with a significantly simpler and more accessible instrumentation platform. The main limitation of the collagen digestion approach is its irreversible nature, which prevents further cultivation or repeated measurements of the same organoid. Nevertheless, for endpoint studies focusing on high-resolution functional imaging, this limitation is acceptable and outweighed by the gain in image quality.

A limitation of the present study is the absence of a fully quantitative calibration of the radioluminescence signal. Although calibration experiments were performed, the derived activity estimates are subject to uncertainties related to sample geometry, cell adhesion, and spatial distribution of the radiotracer. As a result, the reported activity values should be interpreted as semi-quantitative measures rather than absolute quantities.

In particular, a direct conversion to activity per unit area (e.g., Bq/mm^2^) is not straightforward in radioluminescence microscopy due to positron range and optical spreading effects, which lead to a spatial decoupling between the origin of radioactive decay and the detected signal. Despite these limitations, the presented approach enables reliable relative comparisons of radiotracer uptake within and between organoids under consistent experimental conditions, which is relevant for studies of metabolic heterogeneity.

The observed heterogeneous signal distribution is consistent with spatial variations in metabolic activity within the organoids, although the current implementation does not allow direct attribution of the signal to specific cell populations or viability states. Future integration with additional molecular or viability markers could further enhance the biological interpretation of the radioluminescence signal. The estimated sensitivity and spatial resolution of the presented system are in agreement with previously reported radioluminescence microscopy implementations [14], supporting the validity of the approach despite its simplified and accessible design.

Beyond the technical validation, the presented method opens several perspectives for future applications. The combination of radioluminescence microscopy with fluorescence imaging enables direct comparison of metabolic tracer uptake with structural or molecular markers, as demonstrated in previous organoid studies [17]. Moreover, the setup could be used to investigate the dependence of FDG uptake on extracellular glucose concentration, which is highly relevant in the context of PET imaging and patient-specific glucose levels [13].

In principle, the system could also be extended toward dynamic imaging, provided that sufficient signal-to-noise ratio can be maintained at shorter acquisition times. Such measurements would allow investigation of tracer kinetics and compartmental modeling parameters [18, 19]. Further improvements in sensitivity, for example by using objectives with even higher numerical aperture, would directly support this direction.

From an instrumental perspective, additional extensions such as a motorized scanning stage could enable raster scanning of larger specimens or multiple organoids, allowing systematic and automated studies.

## Conclusion

This study demonstrates that a high-resolution radioluminescence microscopy imaging setup for tumor organoids can be realized on the basis of a conventional optical microscope platform and an optimized sample preparation protocol. The developed approach provides a practical and accessible entry point for integrating functional radionuclide imaging into organoid-based cancer research.

## Data Availability

The datasets generated during the current study are available from the corresponding author upon reasonable request.

## Declarations

### Animal welfare

Not applicable. This study did not involve animals.
